# Re-Potentiation of β-Lactam Antibiotic by Synergistic Combination with Biogenic Copper Oxide Nanocubes against Biofilm Forming Multidrug-Resistant Bacteria

**DOI:** 10.3390/molecules24173055

**Published:** 2019-08-22

**Authors:** Ruby Celsia Arul Selvaraj, Mala Rajendran, Hari Prasath Nagaiah

**Affiliations:** Mepco Schlenk Engineering College, Sivakasi 626005, Tamil Nadu, India

**Keywords:** amoxyclav, burn wound, fibroblasts, *Tamarindus indica*, urinary catheter

## Abstract

Biofilm-associated tissue and device infection is a major threat to therapy. The present work aims to potentiate β-lactam antibiotics with biologically synthesized copper oxide nanoparticles. The synergistic combination of amoxyclav with copper oxide nanoparticles was investigated by checkerboard assay and time-kill assay against bacteria isolated from a burn wound and a urinary catheter. The control of biofilm formation and extracellular polymeric substance production by the synergistic combination was quantified in well plate assay. The effect of copper oxide nanoparticles on the viability of human dermal fibroblasts was evaluated. The minimum inhibitory concentration and minimum bactericidal concentration of amoxyclav were 70 μg/mL and 140 μg/mL, respectively, against *Proteus mirabilis* and 50 μg/mL and 100 μg/mL, respectively, against *Staphylococcus aureus*. The synergistic combination of amoxyclav with copper oxide nanoparticles reduced the minimum inhibitory concentration of amoxyclav by 16-fold against *P. mirabilis* and 32-fold against *S. aureus*. Above 17.5 μg/mL, amoxyclav exhibited additive activity with copper oxide nanoparticles against *P. mirabilis*. The time-kill assay showed the efficacy of the synergistic combination on the complete inhibition of *P. mirabilis* and *S. aureus* within 20 h and 24 h, respectively, whereas amoxyclav and copper oxide nanoparticles did not inhibit *P. mirabilis* and *S. aureus* until 48 h. The synergistic combination of amoxyclav with copper oxide nanoparticles significantly reduced the biofilm formed by *P. mirabilis* and *S. aureus* by 85% and 93%, respectively. The concentration of proteins, carbohydrates, and DNA in extracellular polymeric substances of the biofilm was significantly reduced by the synergistic combination of amoxyclav and copper oxide nanoparticles. The fibroblast cells cultured in the presence of copper oxide nanoparticles showed normal morphology with 99.47% viability. No cytopathic effect was observed. Thus, the study demonstrated the re-potentiation of amoxyclav by copper oxide nanoparticles.

## 1. Introduction

Biofilm is a community of bacteria that survives like multicellular organisms. The biofilm phenotype is different from planktonic cells [1]. Biofilm forms through a complex cascade of events that encapsulate bacteria within self-assembled extracellular polymeric substances (EPS) [2]. Approximately 65% to 95% of biofilm is composed of water. The EPS of biofilm are made up of proteins (≥2%), carbohydrates (1–2%), and DNA (≤1%) [3]. It is a viscous layer that prevents the entry of chemotherapeutic agents, leading to the recalcitrance of bacteria [4]. Bacteria inside the biofilm are resistant to external stress and evade the host immune system [5]. The therapeutic failure of antibiotics in the treatment of tissue and medical device-associated infections is mainly due to persistent biofilm formation. About 80% of recalcitrant infections are due to biofilm [6]. Biofilm-associated tissue infections are the sole cause of nosocomial infections [7], periodontitis [5], tooth decay [8], endocarditis [9], pulmonary infections [10], osteomyelitis [11], cystic fibrosis [12], tuberculosis [13], etc. Implantable and non-implantable medical devices are infected by bacteria. They establish biofilm on devices like contact lenses [14], intravenous catheters [15], breast implants [16], orthopedic implants [17], voice prostheses [18], cardiac valves [19], shunts [20], urinary catheters [15], dialysis units [21], ventricular assisted devices [22], etc. Infections caused by biofilm are responsible for high rates of morbidity and mortality. Biofilm architecture, stages of biofilm formation, and device-associated infection were reviewed by Algburi et al. [23].

Chronic wounds are a silent epidemic affecting millions of people globally. Among chronic wounds, burn wounds deserve special attention because of the extensive damage caused by heat. This type of wound is a major cause of mortality and morbidity [24]. Patients who are hospitalized for long periods void urine through urinary catheters [14], and long-term use of indwelling urinary catheters can cause urinary tract infections [25]. Biofilm formation by uropathogens is the most common cause of persistent infection in the genitourinary tract [26].

The post-antibiotic era is witnessing the co-evolution of pathogens resistant to antibiotics [27]. Even after the discovery of many diverse classes of antibiotics, β-lactam makes up 65% of the antibiotic market [28]. β-lactam antibiotics are widely used for a wide range of treatments, from simple boils and fever to the most complicated and life-threatening diseases, such as pneumonia, gonorrhea, meningitis, etc. [29]. Sales of β-lactam antibiotics amount to about $15 billion [30]. These antibiotics are broad-spectrum agents, which makes them the first choice to ward off infection. Bacteria develop resistance to antibiotics due to their improper use [31].

Bacteria synthesize β-lactamase and cleave β-lactam rings [32]. To overcome this challenge, scientists have used β-lactam antibiotics together with β-lactamase inhibitors, such as clavulanic acid (amoxyclav) [33], sulbactam (ampicillin/sulbactam) [34], and tazobactam (piperacillin/tazobactam) [35]. Amoxicillin combined with the β-lactamase inhibitor clavulanic acid was initially effective against a broad spectrum of bacteria [33]. The clinical efficacy and safety of amoxicillin/clavulanic acid were compared with those of clindamycin in the treatment of odontogenic infections in a phase IV clinical trial [36,37]. The study reported that amoxyclav was not inferior to clindamycin in its efficacy and safety. A study by Assimakopoulos et al. [34] evaluated the clinical efficacy of ampicillin/sulbactam in patients in the intensive care unit suffering from ventilator-associated pneumonia caused by pandrug-resistant *Acinetobacter baumannii*. The study concluded that the combination of ampicillin and sulbactam was highly effective against pneumonia [34]. Shirley [36] examined the clinical efficacy of ceftazidime/avibactam and meropenem for the treatment of hospital-acquired and ventilator-associated bacterial pneumonia in a phase III clinical trial and demonstrated the clinical success of ceftazidime/avibactam. However, after a few years of extensive use, bacteria developed recalcitrance to β-lactamase inhibitors (clavulinic acid, sulbactam, avibactam, and tazobactam), as these inhibitors have an integral β-lactam ring, which is also cleaved by β-lactamase, resisting their combination. The prevalence of multidrug resistance among bacteria is due to the single target and single mode of action of antibiotics [33]. The rapid spread of multidrug resistance is due to the horizontal transfer of resistance genes between bacteria. Biofilm is the community of heterogeneous organisms where gene transfer is very easy [38].

One way to circumvent this phenomenon is to re-potentiate the antibiotics by using synergistic combinations with other antibiotics or with natural phytochemicals, such as thymoquinone, a benzoquinone [39] or pterostilbene, a polyphenol [40], which possess medicinal properties. The synergistic combination of more than one drug extends the sensitivity of pathogens to antimicrobial agents, reduces the toxicity of high doses of any single agent, and reduces the chance of developing resistance by acting on multiple targets of bacteria. Recently, metal oxide nanoparticles, such as silver [41] and copper [42], have been used to augment the efficiency of antibiotics. Copper oxide nanoparticles (CuO NPs) are biocompatible and effective against pathogens. There are many methods of synthesizing CuO NPs, such as microwave irradiation [43], sonochemical irradiation [44], etc. Among the many methods available for synthesizing copper nanocubes, green synthesis employs water and does not require any toxic solvents [45]. Green methods for the synthesis of CuO NPs use bacteria [46], fungi [47], algae [48], or plants [49]. Among the plants, *Tamarindus indica* L. (Indian tamarind) fruit possesses many pharmacologically important properties and are used to control infection, fever, diarrhea, jaundice, etc. 

The present work reports the isolation and identification of bacteria from a burn wound and a urinary catheter. The resistance profiles of the bacteria are evaluated and the possibility of synergistically using amoxyclav with CuO NPs is investigated for the ability to mitigate biofilm formation by targeting EPS formation. Moreover, the minimum inhibitory concentration (MIC) and minimum bactericidal concentration (MBC) of the *T. indica* fruit extract, amoxyclav, and CuO NPs are assessed against the isolated bacteria. The synergistic combination of amoxyclav and CuO NPs is investigated by the checkerboard method and a time-kill assay. In addition to the effect on planktonic cells, the effect on biofilm formation and protective EPSs is also investigated in the present study. Finally, the effect of CuO NPs on fibroblast viability is assessed. 

## 2. Results

### 2.1. Isolation, Identification, and Antibiotic Sensitivity of Bacteria from Urinary Catheter and Burn Wound 

**Figure 1:**
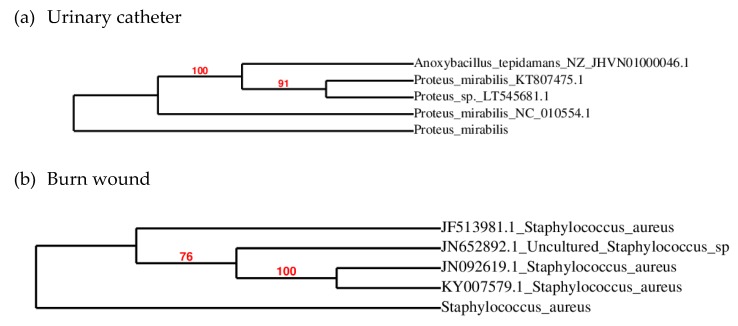
Phylogenetic tree of the bacterial isolate. (**a**) Urinary catheter; (**b**) burn wound.

The main goal of the present study is to evaluate the synergistic activity of CuO NPs with amoxyclav against multidrug-resistant bacteria. Quality control strains are susceptible to all antibiotics and do not reflect the antibiotic-resistant pattern of bacteria prevalent in clinical units. So bacteria were isolated from a burn wound and a urinary catheter of a hospitalized patient in Sivakasi, India’s fireworks headquarters. The bacteria from the urinary catheter and burn wound were identified by 16S ribosomal RNA(rRNA) sequencing as *Proteus mirabilis* and *Staphylococcus aureus*, as shown in Figure 1. 

### 2.2. Susceptibility of Isolates to Antibiotics

Investigating the sensitivity of isolates to antibiotics is essential for proper antibiotic therapy. The susceptibility of the isolated bacteria to different classes of antibiotics was assessed, and the results are presented in Table 1. The lowest susceptibility to amoxicillin (β-lactam class) was shown by *S. aureus*, with an inhibition zone of 3 ± 0.03 mm. Similarly, *P. mirabilis* exhibited very low susceptibility to amoxicillin. When the combination of amoxicillin with clavulanic acid (amoxyclav) was used, the inhibition zone was increased to 10 ± 0.05 mm and 12 ± 0.08 mm against *P. mirabilis* and *S. aureus*, respectively. Even though the inhibition zone was greater than with amoxicillin alone, it was still within the resistance zone as per the Clinical and Laboratory Standards Institute [50]. The highest inhibition was exhibited by gentamicin (aminoglycoside), with an inhibition zone of 25 ± 0.07 mm and 28 ± 0.08 mm with *P. mirabilis* and *S. aureus*, respectively. The inhibition zone shown by azithromycin (macrolide) was 8 ± 0.04 mm and 12 ± 0.07 mm against *P. mirabilis* and *S. aureus*, respectively. The bacteria were designated as susceptible or resistant to an antibiotic based on the guidelines provided by the Clinical and Laboratory Standards Institute [50]. As the bacteria were resistant to more than one class of antibiotic, they were designated as multidrug-resistant. The resistance profile reveals an urgent need to develop strategies to combat multidrug resistance in bacteria.

### 2.3. Synthesis and Characterization of CuO NPs

**Figure 2:**
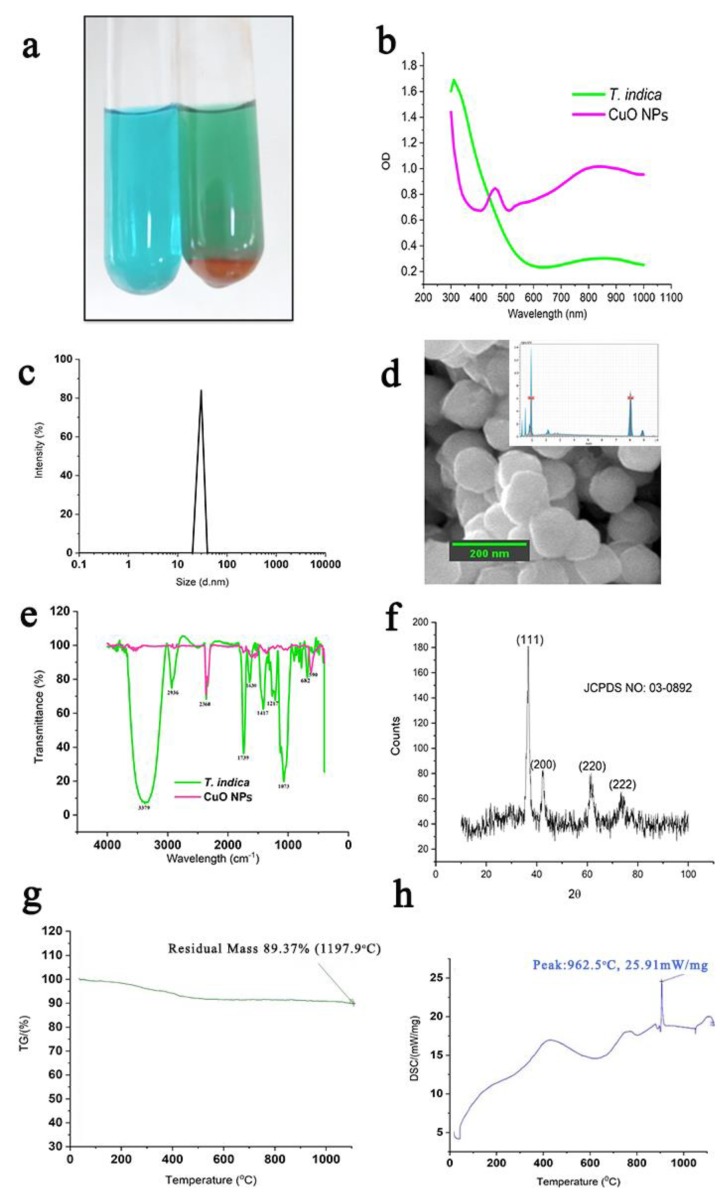
Synthesis and characterization of CuO NPs. (**a**) Formation of CuO NPs; (**b**) UV-Vis spectrum; (**c**) particle size distribution; (**d**) field emission scanning electron microscopic image (FESEM); (**e**) Fourier-transformed infrared spectrum (FTIR); (**f**) X-ray diffraction (XRD); (**g**) differential scanning calorimetry (DSC); (**h**) thermo gravimetric analysis (TGA).

The characteristics of CuO NPs are shown in Figure 2. The formation of CuO NPs (Figure 2a) was initiated by the change from the blue color of copper acetate to green, greenish-yellow, yellowish orange, and finally to the brick-red precipitation of CuO NPs. The UV-Vis absorption spectrum of CuO NPs displayed surface plasmon resonance (SPR) at 490 nm, as shown in Figure 2b. Once the CuO NPs were formed, the size distribution of particles was analyzed in a particle size analyzer, and the result is shown in Figure 2c, showing the homogeneous distribution of particles between 40 nm and 50 nm. The shape of the CuO NPs was observed to be cubes, as shown in Figure 2d. The elemental composition confirms the presence of Cu, as shown in Figure 2d. The participation of various functional groups present in the *T. indica* fruit involved in the synthesis of CuO NPs was recorded by Fourier-transform infrared spectroscopy (FTIR), and the results are shown in Figure 2e. Peaks at 3379 cm^−1^ and 2936 cm^−1^ indicate the stretching vibrations of the OH and CH of the alkyl groups, respectively. The presence of conjugated phenolics is indicated by the peak at 2340 cm^−1^. In the *T. indica* fruit extract, the peak at 1799 cm^−1^ is due to the presence of aromatic structures. The peak observed at 1630 cm^−1^ is due to the stretching vibrations of C=O groups. Peaks at 1417 cm^−1^ and 1207 cm^−1^ are ascribed to CH_2_ bending and C–O–C stretching, respectively. The peak at 682 cm^−1^ represents the presence of halogen-containing compounds. In CuO NPs, the presence of CuO is indicated by the presence of strong stretching vibrations of CuO at 590 cm^−1^. This confirms the metal oxide bond and the monoclinic phase of CuO NPs.It showed a peak at 2340 cm^−1^ that exactly matched the peak of *T. indica*. X-ray diffraction (XRD) shows diffraction peaks at (111), (200), (220), and (222), as shown in Figure 2f. The thermo gravimetric analysis (TGA) curve shown in Figure 2g reveals a loss of 11.37% of the mass. The ultimate reduction in mass indicates a loss of moisture and organic moieties present in the prepared CuO NPs sample. An exothermic peak at 962.5 °C by differential scanning calorimetry (DSC) is an indication of the monoclinic phase of CuO NPs, as shown in Figure 2h.

### 2.4. Antibacterial Activity of CuO NPs

Table 2 shows the concentration-dependent bactericidal activity of CuO NPs from 5 μg/mL to 30 μg/mL. The maximum bactericidal activity of 29 ± 1 mm was observed with 30 μg/mL against *S. aureus. S. aureus* was more susceptible to antimicrobial agents than *P. mirabilis*. In order to understand the antibacterial activity of the *T. indica* fruit extract alone, it was used as control, revealing bactericidal activity with an inhibition zone of 10 ± 0.06 mm and 16 ± 0.06 mm against *P. mirabilis* and *S. aureus*, respectively.

### 2.5. MIC and MBC

Once the bactericidal activity was confirmed by diffusion assay, the minimum concentration required to inhibit bacterial growth was evaluated by determining the minimum inhibitory concentration (MIC) and minimum bactericidal concentration (MBC), and the results are shown in Table 3. As the fruit extract of *T. indica* was used to synthesize CuO NPs, the MIC and MBC of the extract were also quantified. The MIC and MBC of the fruit extract against *P. mirabilis* were 1000 μg/mL and 4000 μg/mL, respectively. The fruit extract had an MIC and MBC of 800 μg/mL and 3200 μg/mL, respectively, against *S. aureus*. The MIC and MBC of amoxyclav were 70 μg/mL and 140 μg/mL, respectively, against *P. mirabilis* and 50 μg/mL and 100 μg/mL, respectively, against *S. aureus*. 

### 2.6. Synergistic Interaction between Amoxyclav and CuO NPs against Bacteria

**Figure 3:**
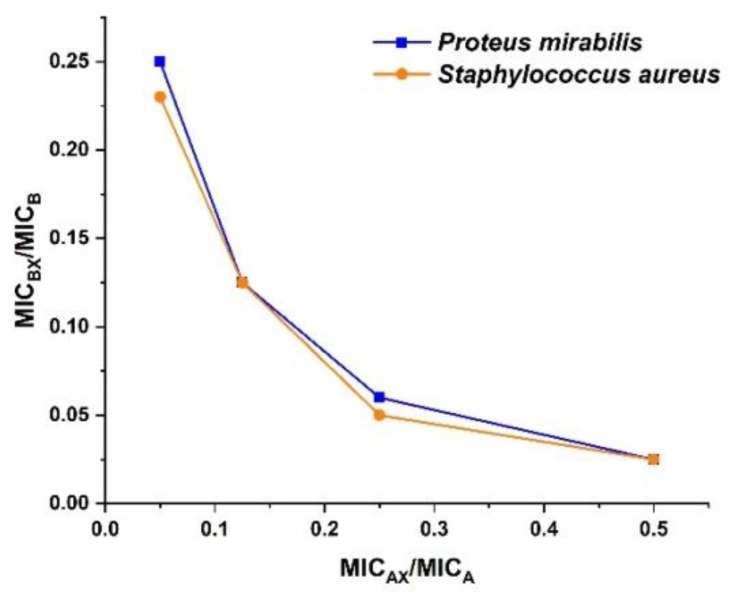
Synergy between amoxyclav and CuO NPs.

The combination of amoxyclav and CuO NPs in different ratios and its impact on fractional inhibitory concentration (FIC) and fractional inhibitory concentration index (FICI) are represented in Table 4. It can be inferred from the table that the FIC of both amoxyclav and CuO NPs decreased significantly at synergistic concentrations. When amoxyclav and CuO NPs were used in synergistic combination, the MIC was reduced 15.9-fold and 2-fold, respectively, for *P. mirabilis*. The synergistic combination reduced the MIC of amoxyclav and CuO NPs 32-fold and 2-fold, respectively, for *S. aureus*. Above 17.5 μg/mL, amoxyclav exhibited additive activity with CuO NPs against *P. mirabilis*. The results obtained from the checkerboard assay proved the synergistic activity of CuO NPs with β-lactam antibiotics through the isobologram depicted in Figure 3. MICA and MICB are the MIC of amoxyclav and CuO NPs, respectively. MICAX and MICBX are the MIC of amoxyclav and CuO NPs in synergistic combination, respectively.

### 2.7. Time-Kill Assay

**Figure 4:**
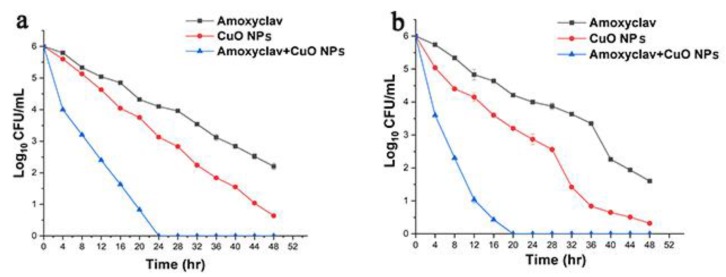
Time-dependent destruction of bacteria by amoxyclav and CuO NPs on (**a**) *P. mirabilis*; and (**b**) *S. aureus.*

Upon determining the synergistic combination of amoxyclav and CuO NPs, the combination was investigated for its time-dependent bactericidal activity. The results of the experiment are shown in Figure 4. With the passing of time from 4 h to 48 h, the reduction in the bacterial population was less with amoxyclav. However, when used in the synergistic combination with CuO NPs, a very significant reduction to 4 log colony forming units (CFU) was observed at 4 h. As the time proceeded to 12 h, it was further reduced to 2.5 log CFU. At 24 h, the bacteria were completely cleared. Amoxyclav and CuO NPs reduced the bacteria to 2.5 log CFU and 0.75 log CFU, respectively. The results demonstrated the effective control of multidrug-resistant pathogens by the synergistic combination of amoxyclav and CuO NPs. The time-kill assay showed the efficacy of the synergistic combination on the complete inhibition of *P. mirabilis* and *S. aureus* within 20 h and 24 h, respectively, whereas amoxyclav and CuO NPs did not inhibit *P. mirabilis* and *S. aureus* until 48 h.

### 2.8. Effect of Amoxyclav and CuO NPs on Biofilm Formation

**Figure 5:**
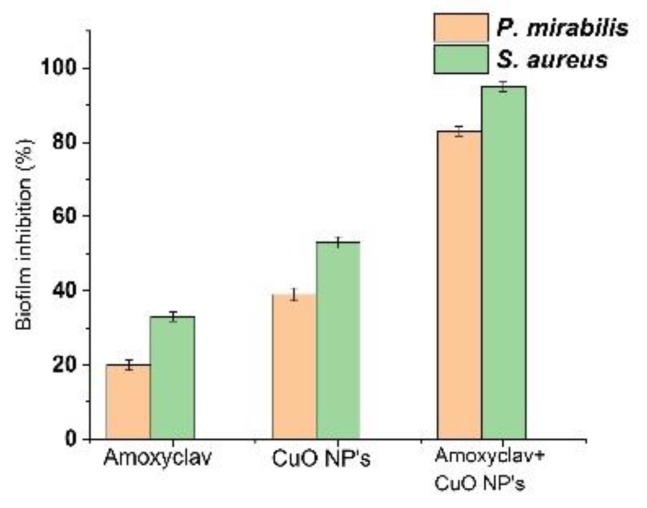
Inhibition of biofilm formation by amoxyclav and CuO NPs.

The synergistic combination of amoxyclav and CuO NPs was assessed for its inhibitory effect on biofilm formation by *P. mirabilis* and *S. aureus*. The results are presented in Figure 5. Amoxyclav inhibited 19% of *P. mirabilis* biofilm and 35% of *S. aureus* biofilm. The synergistic combination of amoxyclav with CuO NPs significantly reduced biofilm formed by *P. mirabilis* and *S. aureus* by 85% and 93%, respectively. Biofilm and planktonic cells of *P. mirabilis* and *S. aureus* showed higher susceptibility to CuO NPs and amoxyclav in synergistic combination. 

### 2.9. Effect of Amoxyclav and CuO NPs on EPS Formation

**Figure 6:**
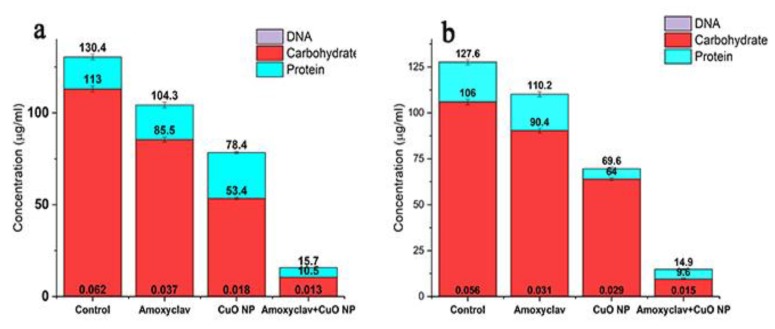
Inhibition of extracellular polymeric substance (EPS) formation by amoxyclav and CuO (**a**) *P. mirabilis;* and (**b**) *S. aureus.*

EPS serves as a barrier to the entry of chemotherapeutic agents. It protects the biofilm community from the host defense. The effect of amoxyclav and CuO NPs on reducing biofilm is shown in Figure 6. In the control, the concentration of proteins was 130.4 µg/mL, which was reduced to 104, 78, and 15.7 µg/mL in the presence of amoxyclav, CuO NPs, and the synergistic combination of amoxyclav and CuO NPs, respectively. In addition to proteins, carbohydrates were also present in EPS. Carbohydrates were remarkably reduced from 113 µg/mL in the control to 10.5 µg/mL in the synergistic combination. Similarly, the concentration of DNA was also reduced in the synergistic combination treatment.

### 2.10. Effect of CuO NPs on Viability of Human Dermal Fibroblasts 

**Figure 7:**
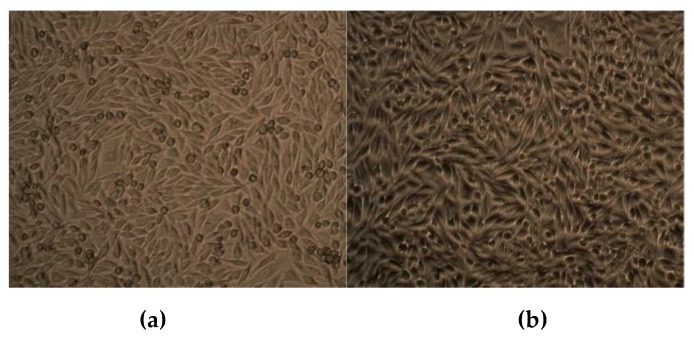
Effect of CuO NPs on the viability of human dermal fibroblasts: (**a**) control; (**b**) CuO NPs.

The effect of CuO NPs on the viability of human dermal fibroblasts (HiFi^TM^ Human Adult Dermal Fibroblast-HiMedia, Mumbai, India) was evaluated, and the results are shown in Table 5 and Figure 7. The viability of the control was 100%. With reference to the control, CuO NPs showed 99.47% viability. The cells cultured in the presence of CuO NPs showed normal morphology and no cytopathic effect was observed. This confirms that CuO NPs are safe for human application. 

## 3. Discussion

**Figure 8:**
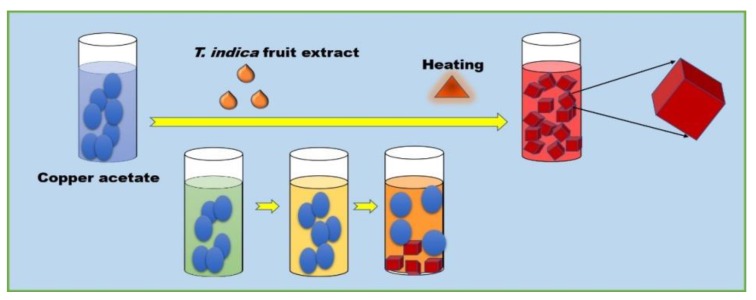
Mechanism of CuO NPs synthesis.

Chronic wounds are infected by different types of pathogens. *S. aureus* is the predominant gram-positive organism reported in burn wounds [51]. *P. mirabilis* and *Escherichia coli* are the dominant agents of urinary tract infections [25]. The present study was focused on re-potentiating amoxyclav by synergistically combining it with CuO NPs due to its multiple modes of action against bacteria. Controlled synthesis of CuO NPs by biological methods was reported [52]. Nanocubes were synthesized at 75 °C within 10 min through a sequence of color changes as shown in Figure 8. During these chemical reactions, copper ions were released from copper acetate and reduced by the reducing groups present in the fruit extract of *T. indica* [51,52]. Red precipitate indicated the formation of CuO NPs [53]. At 70 °C, the reaction was accelerated, leading to the formation of nanocubes. SPR at shorter wavelengths confirmed the smaller size of the CuO NPs [51]. The peak was attributed to the band gap transition of the CuO NPs. The fruit of *T. indica* is a rich source of flavonoids, alkaloids, and many aromatic compounds that are potent antibacterial agents [54]. Studies by Ieven et al. [54] reported that *T. indica* fruit has a higher concentration of secondary metabolites than stem and leaves. These secondary metabolites, when capped onto CuO NPs, can augment the antibacterial activity of the CuO NPs. FTIR data confirmed the presence of various functional groups of compounds in the fruit extract of *T. indica* and CuO NPs synthesized using the fruit. Peaks of conjugated polyphenols were present in both *T. indica* fruit and CuO NPs synthesized using it [55]. The presence of aromatic compounds was confirmed by the FTIR peak. The data indicate that *T. indica* contains a variety of phenolic compounds that play a major role in the formation of CuO NPs. The presence of conjugated phenolics in the extract of *T. indica* was supported by Zaibunnisa, et al. [56]. Polyphenolics such as catechin, epicatechin, taxifolin, apigenin, eriodictyol, luteolin, and naringenin are some of the flavonoids reported to be present in *T. indica* [57]. They serve as stabilizing and capping agents responsible for the well-dispersed synthesis of copper nanocubes. All nanocubes were complete without any truncation. The cubes showed smooth margins and uniform distribution. The process is energetically efficient and does not require extreme purification steps in downstream processing, as it uses no solvent except water.

Ordered cubic-shaped CuO NPs revealed their well-dispersed size and shape. The asymmetric peak of CuO NPs was confirmed by the presence of a peak at 590 cm^–1^ [58]. The high packing efficiency of self-assembled nanocubes was supported in earlier studies [59]. Nanocubes with high packing density are preferable for drug delivery and interaction with cells and biomolecules [60]. The nanocube has a preference for 111 planes over other planes. These results coincide with the observations of Liu et al. [61]. The monoclinic phase of CuO NPs corroborates with the observations of Topnani et al. [58]. Cube-shaped structures provide greater contact area than spherical nanoparticles for interaction with bacteria. The more contact points there are, the greater the bactericidal activity. Studies by Kolhatkar et al., support the greater surface area of nanocubes over other shapes [62]. The thermal properties of CuO NPs were used to show the presence of compounds other than CuO NP.

The miniaturization of CuO at nanoscale provides high surface area and reactivity to interact with bacteria [63]. Phenolic compounds from the fruit of *T. indica* capped the nanocubes, which augment the bactericidal activity of CuO NPs [64]. CuO NPs create oxidative stress and generate reactive oxygen species, which is lethal to pathogens.

The synergistic combination of amoxyclav and CuO NPs significantly reduced biofilm in multiple ways. Amoxyclav is lethal by inhibiting bacterial cell wall biosynthesis. When used in synergistic combination, CuO NPs might bind with β-lactamase, making it ineffective for cleaving the β-lactam ring. In the absence of β-lactamase activity, the potency of amoxyclav was restored. The lowest concentration of amoxyclav and CuO NPs required in the synergistic combination is due to the multiple modes of action of the agents against bacteria. CuO NPs interact with the negatively charged bacterial membrane by electrostatic attraction and accumulate, encapsulating the bacteria [52]. In addition, they impose oxidative stress by forming free radicals, which diffuse freely into the bacteria, causing a lethal effect. Oxidative stress oxidizes proteins, carbohydrates, and nucleic acids [65]. In addition, many different mechanisms, such as the release of copper ions, disruption of membrane integrity, and inhibition of metabolic activity, were proposed for the bactericidal activity of CuO NPs [52]. The synergistic combination was effective against both *P. mirabilis* and *S. aureus*. CuO NPs have a broad spectrum of antibacterial activity against both gram-positive and gram-negative organisms [52]. Complete elimination of biofilm formation by uropathogens was reported for CuO NPs. Similar to the present study, the broad-spectrum bactericidal activity of CuO NPs was documented [66]. CuO NPs were reported to be safe to eukaryotic cells. Studies by De Jong*,* et al. [67] reported that CuO NPs were safe in rats in amounts up to 32 mg/kg. The physiological response of diarrhea was observed in rats only after administration of 512 mg CuO NPs/kg. In the present study, the MIC of CuO NPs was 30 µg/mL, but in the synergistic combination with amoxyclav, the FIC was a maximum of 15 µg/mL. The effect of CuO NPs on fibroblasts was studied at 30 µg/mL, showing 99.47% viability, so the concentration of CuO NPs in synergistic concentration with amoxyclav had no cytotoxic effect.

## 4. Materials and Methods 

### 4.1. Chemicals

All chemicals were purchased from HiMedia, Mumbai, India.

### 4.2. Isolation and Identification of Bacteria

The bacteria infecting the burn wound were isolated by swabbing the wound with a sterile swab before dressing. Immediately the swab was transported in an icebox to the laboratory for the isolation of bacteria. The swab was transferred to a blood agar medium. The plate was incubated for 18 h in an incubator at 37 °C. To determine whether the urinary catheter was also infected, it was collected on the day of replacement and used for the isolation of bacteria. The surface of the catheter was washed with sterile distilled water, and the catheter was opened at cross-sections. The cut catheter samples were placed in buffered saline and incubated in a shaker at 150 rpm (revolutions per minute) for 1 h. Bacteria transferred from the catheter bits to the saline sample were isolated by the pour plate technique. A single colony from the plate was selected and pure cultured. Genomic DNA was isolated using a HiPurA^TM^ Bacterial Genomic DNA Purification Kit, and the 16S rRNA sequence was amplified using the primers 27F 5′-AGAGTTTGATCMTGGCTCAG-3′ and 1492R 5′-TACGGYTACCTTGTTACGACTT-3′. The 16S rRNA sequencing was performed by Yaazh Xenomics, Coimbatore, India. Sequencing reactions were performed using ABI PRISM^®^ BigDye^TM^ Terminator Cycle Sequencing Kits with AmpliTaq^®^ DNA polymerase (FS enzyme) (Applied Biosystems, Thermo Fischer Scientific, Waltham, MA, USA) in an ABI 3730 × l sequencer (Applied Biosystems). Nucleotide sequences were resolved using the basic local alignment search tool on the National Center for Biotechnology Information (NCBI) website and the sequences of related taxa were retrieved. Sequence alignment was conducted and the phylogenetic tree was generated using the neighbor-joining method [68] and Molecular Evolutionary Genetics Analysis (MEGA) version 5.0-Pennsylvania State University, PA, USA [69]. Data analysis was performed on a bootstrapped set with 1000 replicates.

### 4.3. Susceptibility of Bacteria to Antimicrobial Agents

An antibiotic susceptibility test was performed by the disc diffusion method [50]. A single colony of the isolated organisms was inoculated into 2 mL of sterile nutrient broth separately and incubated at 37 °C in a shaker at 120 rpm for 12 h. The culture was adjusted to 1 × 10^6^ CFU/mL, and 100 μL of the culture was swabbed onto Mueller and Hinton agar plates (Himedia, Mumbai, India). Discs of amoxicillin (30 μg), amoxyclav (30 μg), ciprofloxacin (30 μg), cefixime (30 μg), azithromycin (30 μg), and gentamicin (50 μL) were used. The plates were incubated at 37 °C for 12 h, and then the diameter of the zone of inhibition around the well was measured. The zone of inhibition was interpreted using the standards published by the Clinical and Laboratory Standards Institute [50]. 

### 4.4. Synthesis and Characterization of CuO NPs

CuO NPs were synthesized using the aqueous extract of *Tamarindus indica* L. (Indian tamarind) fruit. The *T. indica* fruit was obtained from a local market. The fruit pulp (without seeds) was soaked in water for 60 minutes at room temperature. The aqueous extract of the fruit was prepared by extracting 10 g of fruit pulp with 100 mL of Milli-Q water at room temperature. The extract was centrifuged at 10,000 rpm for 15 min. The supernatant was concentrated and used. The extract was centrifuged at 10,000 rpm for 15 min. The supernatant was concentrated and used. Under heating and constant stirring, 10 mL of extract was slowly added to 100 mL of 3 μΜ copper acetate solution. The red precipitate was separated by centrifugation at 15,000 rpm for 15 min. The precipitate was dried and stored [70]. Visible light absorption and vibrational spectra were recorded using a UV-Vis spectrophotometer (Hitachi, Tokyo, Japan) and Fourier-transform infrared spectrophotometer (Thermo Sceintific Ltd., Waltham, MA, USA). The size of the CuO NPs was documented using a particle size analyzer. Structural and elemental analysis was captured using field emission scanning electron microscopy (FESEM, Carl Zeiss, Cambridge, UK) and energy dispersive spectroscopy (Bruker, Billerica, MA, USA). The crystalline behavior of copper nanoparticles was recorded using a powder X-ray diffractometer (X’Pert Pro–PANalytic-Malvern Panalytical, Almelo, The Netherlands). The thermal stability of CuO NPs was determined using DSC coupled with TGA (Netzch, Selb, Germany).

### 4.5. Determination of Antibacterial Activity, MIC, and MBC

The bactericidal activity of CuO NPs (30 μg) was determined by well diffusion. The MIC and MBC of CuO NPs, and amoxyclavh were determined by 2-fold serial dilution in broth [71]. The experiment was performed in flat-bottomed 96-well plates. The cultures of *P. mirabilis* and *S. aureus* grown overnight were adjusted to 10^8^ CFU/mL. The plate was maintained with broth as a negative control. Growth control was maintained with the broth and the culture without any antimicrobial agents. In one of the wells, 100 μL of amoxyclav (2040 µg/mL) was added. In a separate well, 100 μL of CuO NPs (1920 μg/mL) was added. From these wells, 2-fold dilutions were done serially in the subsequent wells to attain 2040µg /mL to 4.35 μg/mL amoxyclav and 1920 μg/mL to 3.75 μg/mL CuO NPs. Then 100 μL each of *P. mirabilis* and *S. aureus* (10^8^ CFU/mL) was added to the respective plates. The plates were incubated at 37 °C for 12 h. The concentration of the antimicrobial agent in the well where no visible growth was observed was considered as the MIC. Then 100 μL of the sample from the respective MIC wells and the three preceding wells were taken and plated onto nutrient agar to determine the MBC. The concentration of the antimicrobial agent where no colony was found in the plate was recorded as the MBC.

### 4.6. Time-Kill Assay

The synergy between amoxyclav and CuO NPs was determined by a time-kill assay [72]. To determine the time-dependent lethal effect of amoxyclav and CuO NPs on *P. mirabilis* and *S. aureus*, the culture grown overnight was adjusted to 1 × 10^8^ CFU/mL. Then 25 mL of Luria-Bertani (LB) broth was inoculated with 1 mL each of *P. mirabilis* and *S. aureus* in separate conical flasks, and 30 μg/mL each of CuO NPs and amoxyclav were added to the flasks. The flasks were kept in an incubator-cum-shaker at 37 °C. Growth control was maintained in the same manner except for the addition of any antimicrobial agent. Viable colony count was measured by sampling the culture at 4 h intervals for 48 h. Synergistic interaction was determined by the reduction in the number of viable cells by more than 2 logs between the single agent and the combination of agents. A reduction of less than 2 logs indicated no difference.

### 4.7. Synergistic Interaction between Amoxyclav and CuO NPs

The synergistic interaction between two or more drugs was determined by checkerboard assay, where the test compounds were serially diluted alone and in all combinations of drugs [73]. The fractional inhibitory concentration (FIC) of amoxyclav and CuO NPs was determined by 2-fold serial dilution from 0.5 × MIC to 0.015 × MIC. To determine FICI, amoxyclav was varied in a descending manner and CuO NPs were decreased vertically. In the plates, negative control was maintained with the broth and the growth control was maintained with culture alone without any antimicrobial agent for comparison. The wells other than the negative control were loaded with 10 µL of 1 × 10^6^ CFU/mL of *P. mirabilis* and *S. aureus* in separate plates. The plates were incubated at 37 °C for 24 h. The wells where bacterial growth was reduced by more than 80% were considered as the MIC. The combined effect of the drugs was concluded from the FICI value: FICI = MIC in combination/MIC alone of amoxyclav + MIC in combination/MIC alone of CuO NPs. The data were interpreted according to the instructions of the European Committee on Antimicrobial Susceptibility Testing [74]. 

### 4.8. Effect of Amoxyclav and CuO NPs on Biofilm Formation

To evaluate biofilm inhibition by antibiotics and CuO NPs, biofilm was formed in 96-well flat-bottomed polystyrene microtiter plates in triplicate [75]. For this, 10 μL each of (1 × 10^6^ CFU/mL) *P. mirabilis* and *S. aureus* was inoculated onto 190 μL of LB in separate wells, then 10 μL of CuO NPs (30 μg/mL) was added to the wells. Negative control was maintained without an antimicrobial agent and positive control was maintained with amoxyclav and CuO NPs. The plates were incubated at 37 °C for 24 h. The medium was aspirated in order to remove the planktonic cells and gently washed thrice with phosphate-buffered saline. Then 200 μL of 2% crystal violet solution was added to the wells and left undisturbed for 20 min. Excess crystal violet stain was removed and washed using a buffer. The crystal violet staining the biofilm was solubilized by 125 μL of 30% acetic acid. The color was measured using a microplate reader (BIO-RAD, Hercules, CA, USA) at 570 nm.

### 4.9. Effect of Amoxyclav and CuO NP on EPS Formation 

Biofilm was formed in 96-well plates in triplicate, as mentioned above [75]. After biofilm formation, the cells were not stained with crystal violet. Instead, the attached cells were collected by vigorous mixing and washing with a 2 mM phosphate buffer at pH 7. The suspension was vortexed for 30 min and an equal volume of 30% ethylenediaminetetraacetic acid (EDTA) was added. The mixture was incubated in a shaker for 3 h at 4 °C. The sample was centrifuged at 15,000 rpm for 15 min. The supernatant was filtered, sterilized (0.22 μm), and dialyzed in a dialysis bag with a 3500 Da cutoff against Milli-Q water to separate the high-molecular-mass compounds. The sample was used for the quantification of carbohydrates, proteins, and DNA. The total carbohydrates were quantified by the phenol–sulfuric acid method [76]. Proteins were determined by Lowry’s method [77]. DNA was quantified by the dye-binding method [78]. 

## 5. Effect of CuO NPs on Viability of Human Dermal Fibroblasts

The effect of CuO NPs on the viability of fibroblasts was quantified by (3-[4, methylthiazol-2-yl]-2, 5-diphenyl tetrazolium bromide (MTT) dye assay [79]. This test is based on the reduction of water-soluble yellow MTT dye to water-insoluble purple formazan crystals by live cells. The cells (500 µL) were seeded onto wells at a density of 1 × 10^6^ cells/mL in Dulbecco’s modified eagle medium supplemented with 10% fetal bovine serum. The plates were incubated in a humidified CO_2_ incubator maintained at 37 °C for 24 h. After 24 h, 30 µg of CuO NPs was added to the wells. A control well was maintained without CuO NPs. After 48 h, the medium was removed and replaced with a fresh medium containing MTT (5 mg/mL) and incubated in the dark for 4 h. The purple formazan crystals were dissolved in dimethylsulfoxide and read at 570 nm in an ELISA reader (Thermo Sceintific Ltd., Waltham, MA, USA). The viability of cells was calculated with reference to the absorbance of control cells using the formula:

Viability (%) = (Control OD)/(Test OD) ×100

## Figures and Tables

**Table 1:** Antibiotic sensitivity profiles of bacteria.

Diameter of Inhibition Zone (mm)							
S. No	Bacteria	Amoxicillin	Amoxyclav	Cefixime	Ciprofloxacin	Gentamicin	Azithromycin
1	*P. mirabilis*	5 ± 0.02	10 ± 0.05	11 ± 0.09	13 ± 1	25 ± 0.07	8 ± 0.04
2	*S. aureus*	3 ± 0.03	12 ± 0.08	12 ± 0.05	14 ± 0.06	28 ± 0.08	12 ± 0.07

**Table 2:** Antibacterial activity of CuO NPs (diameter of inhibition zone in mm).

Concentration of CuO NPs (μg/mL)							
S. No.	Bacteria	5	10	15	20	25	30
1	*P. mirabilis*	5	10 ± 0.06	14 ± 0.08	16± 0.05	18 ± 1	24 ± 1
2	*S. aureus*	8	14 ± 0.07	18 ± 0.07	21± 0.09	24 ± 1	29 ± 1
Concentration of T. indica fruit extract (30 μg/mL)							
1	*P. mirabilis*	10 ± 0.06					
2	*S. aureus*	16 ± 0.06					

**Table 3:** Antibacterial activity of amoxyclav and CuO NPs.

*P. mirabilis*					*S. aureus*		
S. No.	Antibacterial Activity	Amoxyclav	CuO NP	*T. indica* Fruit Extract	Amoxyclav	CuO NP	*T. indica* Fruit Extract
1	MIC (μg/mL)	70	30	1000	50	20	800
2	MBC (μg/mL)	140	60	4000	100	40	3200

**Table 4:** Fractional inhibitory concentration index (FICI) of amoxyclav and CuO NPs.

*P. mirabilis*					
Amoxyclav (μg/mL)	CuO NP (μg/mL)	FIC of Amoxyclav	FIC of CuO NP	FICI	Interaction
4.4	15	0.062	0.25	0.267	Synergistic
8.8	7.5	0.125	0.126	0.251	Synergistic
17.5	3.8	0.25	0.063	0.313	Synergistic
35	1.9	0.5	0.031	0.531	Additive
***S. aureus***					
1.56	10	0.03	0.5	0.503	Additive
3.15	5	0.06	0.25	0.31	Synergistic
6.25	2.5	0.12	0.125	0.225	Synergistic
12.5	1.25	0.24	0.06	0.31	Synergistic

**Table 5:** Impact of CuO NPs on viability of human dermal fibroblast cells.

S. No.	Treatment	Viability (%)
1	Control	100
2	CuO NPs (30 μg/mL)	99.47
